# Supplementary material for: HIVprotI: an integrated web based platform for prediction and design of HIV proteins inhibitors
Source: J Cheminform. 2018 Mar 9;10:12. doi: 10.1186/s13321-018-0266-y (PMC5845081; doi:10.1186/s13321-018-0266-y)
Supplement: Supplementary file 2 — Additional file 2. Source code of HIVProtI web server. [file 13321_2018_266_MOESM2_ESM.zip › HIVprotI_Source-code/hivprotI/weka-3-6-6/doc/weka/classifiers/trees/j48/NBTreeSplit.html]

NBTreeSplit


---


|  |  |  |
| --- | --- | --- |
| **Overview** \| **Package** \| **Class** \| **Tree** \| **Deprecated** \| **Index** \| **Help** \| **Weka's home** | |  |
| **PREV CLASS**   **NEXT CLASS** | **FRAMES**    **NO FRAMES**     **All Classes** |  |
| SUMMARY: NESTED \| FIELD \| CONSTR \| METHOD | DETAIL: FIELD \| CONSTR \| METHOD |  |


---


## weka.classifiers.trees.j48 Class NBTreeSplit

```
java.lang.Object
  weka.classifiers.trees.j48.ClassifierSplitModel
      weka.classifiers.trees.j48.NBTreeSplit
```

**All Implemented Interfaces:**: java.io.Serializable, java.lang.Cloneable, RevisionHandler

---

``` public class NBTreeSplit extends ClassifierSplitModel ```

Class implementing a NBTree split on an attribute.

**Version:**
:   $Revision: 1.5 $

**Author:**
:   Mark Hall (mhall@cs.waikato.ac.nz)

**See Also:**: Serialized Form

---

**Constructor Summary**

`NBTreeSplit(int attIndex,
int minNoObj,
double sumOfWeights)`
  
          Initializes the split model.


| **Method Summary** | |
| --- | --- |
| `int` | `attIndex()`             Returns index of attribute for which split was generated. |
| `void` | `buildClassifier(Instances trainInstances)`             Creates a NBTree-type split on the given data. |
| `double` | `classProb(int classIndex, Instance instance, int theSubset)`             Return the probability for a class value |
| `double` | `getErrors()`             Return the errors made by the naive bayes models arising from this split. |
| `NBTreeNoSplit` | `getGlobalModel()`             Return the global naive bayes model for this node |
| `java.lang.String` | `getRevision()`             Returns the revision string. |
| `java.lang.String` | `leftSide(Instances data)`             Prints left side of condition.. |
| `java.lang.String` | `rightSide(int index, Instances data)`             Prints the condition satisfied by instances in a subset. |
| `void` | `setGlobalModel(NBTreeNoSplit global)`             Set the global naive bayes model for this node |
| `java.lang.String` | `sourceExpression(int index, Instances data)`             Returns a string containing java source code equivalent to the test made at this node. |
| `double[]` | `weights(Instance instance)`             Returns weights if instance is assigned to more than one subset. |
| `int` | `whichSubset(Instance instance)`             Returns index of subset instance is assigned to. |

**Methods inherited from class weka.classifiers.trees.j48.ClassifierSplitModel**

`checkModel, classifyInstance, classProbLaplace, clone, codingCost, distribution, dumpLabel, dumpModel, numSubsets, resetDistribution, sourceClass, split`

**Methods inherited from class java.lang.Object**

`equals, getClass, hashCode, notify, notifyAll, toString, wait, wait, wait`

**Constructor Detail**

### NBTreeSplit

```
public NBTreeSplit(int attIndex,
                   int minNoObj,
                   double sumOfWeights)
```

:   Initializes the split model.


**Method Detail**

### buildClassifier

```
public void buildClassifier(Instances trainInstances)
                     throws java.lang.Exception
```

:   Creates a NBTree-type split on the given data. Assumes that none of
    the class values is missing.

    :   **Specified by:**: `buildClassifier` in class `ClassifierSplitModel`
    :   **Throws:**: `java.lang.Exception` - if something goes wrong

---


### attIndex

```
public final int attIndex()
```

:   Returns index of attribute for which split was generated.

---


### whichSubset

```
public final int whichSubset(Instance instance)
                      throws java.lang.Exception
```

:   Returns index of subset instance is assigned to.
    Returns -1 if instance is assigned to more than one subset.

    :   **Specified by:**: `whichSubset` in class `ClassifierSplitModel`
    :   **Throws:**: `java.lang.Exception` - if something goes wrong

---


### weights

```
public final double[] weights(Instance instance)
```

:   Returns weights if instance is assigned to more than one subset.
    Returns null if instance is only assigned to one subset.

    :   **Specified by:**: `weights` in class `ClassifierSplitModel`

---


### sourceExpression

```
public final java.lang.String sourceExpression(int index,
                                               Instances data)
```

:   Returns a string containing java source code equivalent to the test
    made at this node. The instance being tested is called "i".

    :   **Specified by:**: `sourceExpression` in class `ClassifierSplitModel`
    :   **Parameters:**: `index` - index of the nominal value tested: `data` - the data containing instance structure info **Returns:**: a value of type 'String'

---


### rightSide

```
public final java.lang.String rightSide(int index,
                                        Instances data)
```

:   Prints the condition satisfied by instances in a subset.

    :   **Specified by:**: `rightSide` in class `ClassifierSplitModel`
    :   **Parameters:**: `index` - of subset: `data` - training set.

---


### leftSide

```
public final java.lang.String leftSide(Instances data)
```

:   Prints left side of condition..

    :   **Specified by:**: `leftSide` in class `ClassifierSplitModel`
    :   **Parameters:**: `data` - training set.

---


### classProb

```
public double classProb(int classIndex,
                        Instance instance,
                        int theSubset)
                 throws java.lang.Exception
```

:   Return the probability for a class value

    :   **Overrides:**: `classProb` in class `ClassifierSplitModel`
    :   **Parameters:**: `classIndex` - the index of the class value: `instance` - the instance to generate a probability for: `theSubset` - the subset to consider **Returns:**: a probability **Throws:**: `java.lang.Exception` - if an error occurs

---


### getGlobalModel

```
public NBTreeNoSplit getGlobalModel()
```

:   Return the global naive bayes model for this node

    :   **Returns:**: a `NBTreeNoSplit` value

---


### setGlobalModel

```
public void setGlobalModel(NBTreeNoSplit global)
```

:   Set the global naive bayes model for this node

    :   **Parameters:**: `global` - a `NBTreeNoSplit` value

---


### getErrors

```
public double getErrors()
```

:   Return the errors made by the naive bayes models arising
    from this split.

    :   **Returns:**: a `double` value

---


### getRevision

```
public java.lang.String getRevision()
```

:   Returns the revision string.

    :   **Returns:**: the revision


---


|  |  |  |
| --- | --- | --- |
| **Overview** \| **Package** \| **Class** \| **Tree** \| **Deprecated** \| **Index** \| **Help** \| **Weka's home** | |  |
| **PREV CLASS**   **NEXT CLASS** | **FRAMES**    **NO FRAMES**     **All Classes** |  |
| SUMMARY: NESTED \| FIELD \| CONSTR \| METHOD | DETAIL: FIELD \| CONSTR \| METHOD |  |


---
